# Transcription Factors Runx1 and Runx3 Suppress Keratin Expression in Undifferentiated Keratinocytes

**DOI:** 10.3390/ijms231710039

**Published:** 2022-09-02

**Authors:** Eisaku Ogawa, Tomohiro Edamitsu, Hidetaka Ohmori, Kazuyoshi Kohu, Mineo Kurokawa, Hiroshi Kiyonari, Masanobu Satake, Ryuhei Okuyama

**Affiliations:** 1 Department of Dermatology, Shinshu University School of Medicine, Matsumoto 390-8621, Japan; 2 Department of Molecular Immunology, Institute of Development, Aging and Cancer, Tohoku University, Sendai 980-8575, Japan; 3 Department of Hematology and Oncology, Graduate School of Medicine, The University of Tokyo, Tokyo 113-0033, Japan; 4 Laboratory for Animal Resources and Genetic Engineering, RIKEN Center for Biosystems Dynamics Research, Kobe 650-0047, Japan

**Keywords:** runt-related transcription factor (Runx), keratinocyte, differentiation, proliferation

## Abstract

The Runt-related transcription factor (Runx) family has been suggested to play roles in stem cell regulation, tissue development, and oncogenesis in various tissues/organs. In this study, we investigated the possible functions of Runx1 and Runx3 in keratinocyte differentiation. Both Runx1 and Runx3 proteins were detected in primary cultures of mouse keratinocytes. Proteins were localized in the nuclei of undifferentiated keratinocytes but translocated to the cytoplasm of differentiated cells. The siRNA-mediated inhibition of *Runx1* and *Runx3* expression increased expression of keratin 1 and keratin 10, which are early differentiation markers of keratinocytes. In contrast, overexpression of Runx1 and Runx3 suppressed keratin 1 and keratin 10 expression. Endogenous Runx1 and Runx3 proteins were associated with the promoter sequences of *keratin 1* and *keratin 10* genes in undifferentiated but not differentiated keratinocytes. In mouse skin, the inhibition of *Runx1* and *Runx3* expression by keratinocyte-specific gene targeting increased the ratios of keratin 1- and keratin 10-positive cells in the basal layer of the epidermis. On the other hand, inhibition of *Runx1* and *Runx3* expression did not alter the proliferation capacity of cultured or epidermal keratinocytes. These results suggest that Runx1 and Runx3 likely function to directly inhibit differentiation-induced expression of *keratin 1* and *keratin 10* genes but are not involved in the regulation of keratinocyte proliferation.

## 1. Introduction

The skin is the boundary that separates the inside of the body from the outside world and serves as a physical and immunological barrier [1]. The physical barrier is primarily mediated by the epidermis, which is composed mainly of keratinocytes and is divided into the basal layer at the bottom, the spinous and granular middle layers, and the stratum corneum on the surface. Keratinocytes proliferate in the basal layer, migrate through differentiation, and eventually lose their nuclei to form the stratum corneum.

The proliferation, differentiation, and pathology of keratinocytes have been investigated extensively [2,3]. Inflammatory diseases, such as psoriasis, and cutaneous malignancies, such as squamous cell carcinoma, exhibit significant abnormalities in the regulation of keratinocyte proliferation and differentiation [4,5]. Various molecules are involved in keratinocyte regulation. For example, the transcription factor p63 is essential for keratinocytes to form the epidermis [6]. Mice with deficiency of *p63* gene expression in the skin die soon after birth due to the lack of skin formation [7,8]. At the cellular level, we and other groups reported that p63 is expressed in the basal layer of epidermal keratinocytes and functions to maintain the proliferation potential of immature keratinocytes and blocks keratinocyte differentiation by counteracting the signaling activity of the Notch receptor [9,10]. Furthermore, we reported that p63 inhibits expression of filaggrin and loricrin, markers of differentiated keratinocytes [11], whereas others reported that p63 induces expression of keratin 14, a marker of immature keratinocytes [12]. It is also possible that p63 directly regulates transcription of *NOTCH1*, as p63 was shown to bind to the promoter of this gene [13]. With regard to the interplay between transcription factors, p63 was reported to suppress *HES-1* gene expression [9] and activate transcription of the *Runx1* gene [14].

The Runt-related transcription factors (Runx) are involved in various signaling pathways and cellular processes through multiple protein interactions [15]. The Runx family consists of three members in mammals: Runx1, Runx2, and Runx3. In mouse models, disruption of individual *Runx* genes results in distinct phenotypes that indicate tissue-specific and non-overlapping roles of each *Runx* member in various developmental processes [16,17,18,19,20]. In carcinogenesis, *Runx1* is involved in leukemogenesis [16], whereas *Runx3* is often inactivated in several types of cancer [15].

In this study, we examined the expression and function of Runx1 and Runx3 transcription factors in keratinocyte regulation using primary cultures of mouse keratinocytes and mouse skin. Our results showed that Runx1 and Runx3 most likely function to directly inhibit differentiation-induced *keratin 1* and *keratin 10* gene expression but are not involved in the regulation of keratinocyte proliferation.

## 2. Result

### 2.1. Both Runx1 and Runx3 Transcripts and Respective Proteins Were Expressed in Cultured Keratinocytes

**Figure 1:**
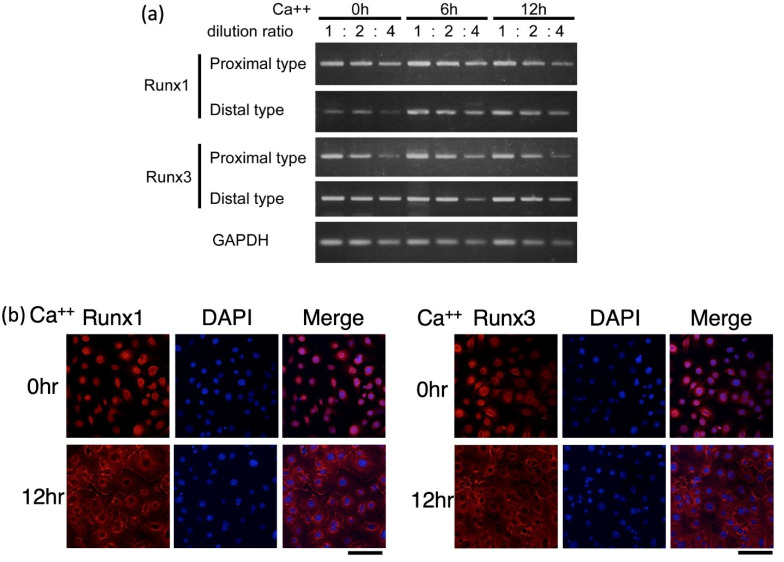
Expression of *Runx1/Runx3* transcripts (**a**) and proteins (**b**) in cultured keratinocytes. The differentiation of keratinocytes was induced by increasing the calcium concentration in the medium for 0, 6, and 12 h of incubation as indicated. (**a**) RNA was extracted from the cells and processed for cDNA synthesis. Relative amounts of proximal and distal *Runx1/Runx3* transcripts were evaluated by semi-quantitative PCR using the 1-, 2-, and 4-fold diluted cDNAs. *GAPDH* transcript was used as a control. The primers that were set to distinguish proximal versus distal type of transcripts yielded amplified products of rather long size. Since this made real time PCR experiments difficult to do, semi-quantitative PCR was chosen. Note that the purpose of experiments here was to confirm the presence of *Runx* transcripts but not try to measure the exact variation in transcript amounts. (**b**) Cells were fixed and immunostained for Runx1/Runx3 protein. DAPI was used to stain nuclei. Scale bar, 100 μm. In both (**a**,**b**) we performed three independent trials for each experimental set out and could obtain essentially similar results. In the figure, representative results are shown.

First, we examined whether *Runx1* and/or *Runx3* were expressed in keratinocytes. We used primary cultures of mouse keratinocytes. The cells cultured in a low-calcium environment mimicked immature proliferating keratinocytes, while those cultured under high-calcium conditions mimicked differentiating keratinocytes [21]. The levels of *Runx* transcripts were evaluated by semi-quantitative reverse transcription (RT)-PCR analysis. As *Runx1* and *Runx3* are transcribed from two different promoters, transcripts of proximal type and those of distal type were measured separately (Figure 1a). The keratinocytes were first cultured in low-calcium medium and then induced to differentiate by culturing in high-calcium medium for 0, 6, and 12 h. Both proximal and distal transcripts were detected for *Runx1* and *Runx3* before and after the induction of differentiation.

Next, we examined Runx1 and/or Runx3 expression at the protein level. The keratinocytes were processed for immunostaining using specific antibodies for Runx1 and Runx3 (Figure 1b). Both Runx1 and Runx3 proteins were detected in the nuclei of undifferentiated keratinocytes (see 0 h). As 4′,6-diamidino-2-phenylindole (DAPI) stained the nuclei, overlap of Runx and DAPI staining indicated the nuclear localization of each Runx protein. Interestingly, in the differentiation-induced keratinocytes (see 12 h), both Runx1 and Runx3 proteins were detected in the cytoplasm but not in the nuclei. The mechanism of nuclear-to-cytoplasmic translocation and putative function of cytoplasmic Runx protein are not yet known. Within the limitations of the present consensus on Runx protein function, the results suggested that Runx protein may act as a transcription factor in undifferentiated but not differentiated keratinocytes.

### 2.2. Inhibition of Runx1 and Runx3 Expression Increased Keratin 1 and Keratin 10 Expression in Cultured Keratinocytes

**Figure 2:**
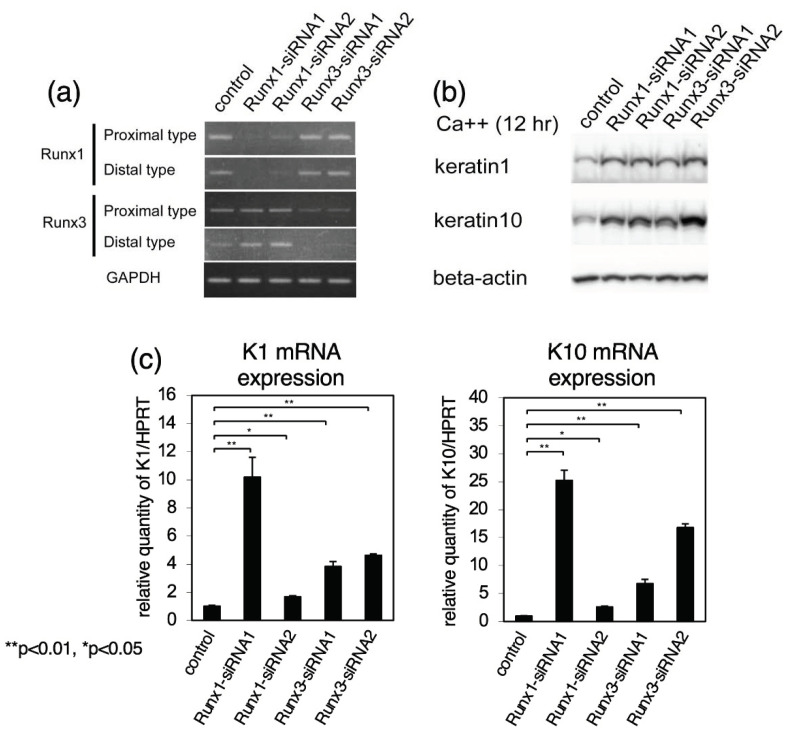
Effects of inhibition of *Runx1*/*Runx3* expression on *keratin 1*/*keratin 10* mRNA and protein expression in cultured keratinocytes. The cells were transfected with specific siRNA for *Runx1* or *Runx3*, incubated for 4 h, induced to differentiate by raising the calcium concentration for 12 h, and harvested. Two different sequences were used for *Runx1* and *Runx3* siRNA, shown as *siRNA1* and *siRNA2*. (**a**) Effects of *Runx1*/*Runx3* siRNAs on *Runx1*/*Runx3* expression. RNA was prepared from the cells and processed for RT-PCR. Proximal and distal *Runx1*/*Runx3* transcripts were examined with *GAPDH* transcript as a control. (**b**) Effects of *Runx1*/*Runx3* siRNAs on keratin 1 and keratin 10 protein expression. Protein lysates were prepared from the cells and processed for immunoblotting analysis of keratin 1, keratin 10, and β-actin (control). (**c**) Effects of *Runx1*/*Runx3* siRNAs on *keratin 1* and *keratin 10* gene expression. RNA was prepared from the cells and processed for real-time RT-PCR analysis. The levels of *keratin 1* and *keratin 10* transcripts normalized relative to *HPRT* transcript are shown. In each experimental set out as above, we performed three independent trials and could obtain essentially similar results. In (**a**,**b**), representative results are shown, whereas in (**c**), mean ± standard deviation are shown, and statistically significant differences, if detected, are indicated by brackets (a student *t*-test; * *p* < 0.05, ** *p* < 0.01).

Keratin 1 and keratin 10 are keratinocyte differentiation markers that are induced during cell differentiation. As Runx1/Runx3 were found to be expressed in the nuclei of undifferentiated keratinocytes, we examined the effects of *Runx* inhibition on keratin 1 and keratin 10 expression. As shown in Figure 2a, we introduced small interfering RNAs (siRNAs) for *Runx1* or *Runx3* into the cells. In the *Runx1* siRNA-introduced keratinocytes, the levels of *Runx1* (but not *Runx3*) transcripts were markedly decreased, whereas in the *Runx3* siRNA-introduced keratinocytes, the levels of *Runx3* (but not *Runx1*) transcripts were markedly decreased. We also confirmed that the level of Runx protein was remarkably reduced in the si-RNA-treated cells (see Supplemental Figure S1). Keratinocytes were then cultured in high-calcium medium for 12 h and cell lysates were processed for immunoblotting analysis of keratin protein expression (Figure 2b). The levels of keratin 1 and keratin 10 expression were substantially increased in siRNA-treated keratinocytes in comparison to cells treated with nonspecific control siRNA. SiRNA-treatment and the following immunoblot were performed three times and the band densities were measured. The levels of keratin 1 and keratin 10 proteins normalized relative to beta-actin are shown as mean and SD (Supplemental Figure S2). Cell lysates were also processed for RT-qPCR analysis of keratin transcripts (Figure 2c). Although the effects were variable between siRNAs, the levels of *keratin 1* and *keratin 10* transcripts were increased in keratinocytes treated with each siRNA. Therefore, the data shown in Figure 2 indicated that inhibition of *Runx1* and *Runx3* expression increased expression of keratin 1 and keratin 10 in differentiating keratinocytes.

### 2.3. Overexpression of Runx1 and Runx3 Suppressed Keratin 1 and Keratin 10 Expression in Cultured Keratinocytes

**Figure 3:**
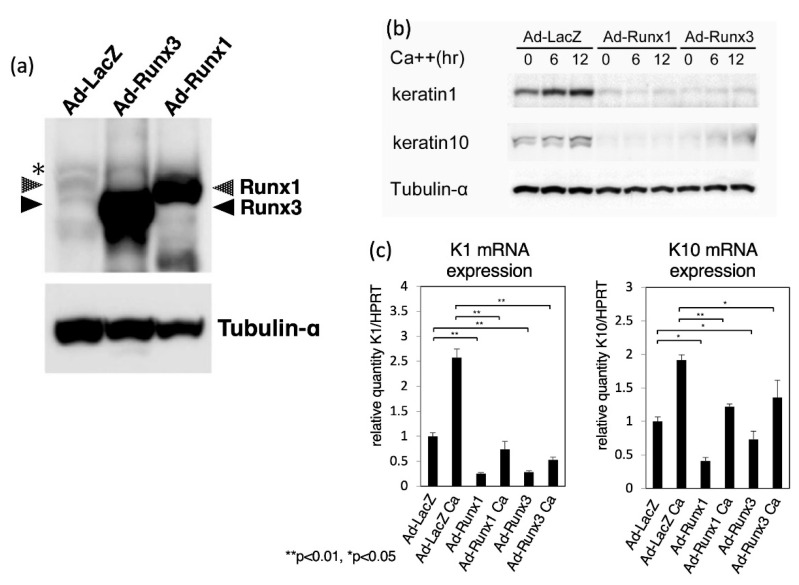
Effects of Runx1/Runx3 overexpression on *keratin 1*/*keratin 10* transcript and protein levels in cultured keratinocytes. Cells were infected with adenovirus vectors carrying LacZ (control), Runx1, or Runx3 for 24 h and cell differentiation was induced by increasing the calcium concentration for 0, 6, and 12 h in (**b**) and for 12 h in (**c**). (**a**) Protein lysates were prepared from the cells and processed for immunoblotting analysis of Runx1, Runx3, and α-tubulin (control). (**b**) Protein lysates were prepared from the cells and processed for immunoblotting analysis of keratin 1, keratin 10, and α-tubulin (control). (**c**) RNA was prepared from the cells and processed for cDNA synthesis and real-time PCR analysis. The levels of *keratin 1* and *keratin 10* transcripts normalized relative to *HPRT* transcript are shown. In each experimental set out as above, we performed three independent trials and could obtain essentially similar results. In (**a**) and (**b**) representative results are shown, whereas in (**c**) mean ± standard deviation are shown and statistically significant differences, if detected, are indicated by brackets (a student *t*-test; * *p* < 0.05, ** *p* < 0.01). Note that the image in (**a**) is extensively magnified so that the endogenous Runx bands are visible. The bands indicated by an asterisk are due to a nonspecific reaction.

The results shown in Figure 2 suggest that Runx1 and Runx3 likely function to suppress expression of keratin 1 and keratin 10. Therefore, we examined the effects of Runx1/Runx3 overexpression on the expression of keratin 1 and keratin 10. As shown in Figure 3a, expression of exogenously introduced Runx1/Runx3 protein in cultured keratinocytes was confirmed by immunoblotting analysis. Note that endogenous Runx proteins were detected as faint bands in the lane of Ad-LacZ and that anti-panRunx antibody was used in Figure 3a (As for the immunofluorescent detection of overexpressed Runx proteins, see Supplemental Figure S3). Then, these cells were cultured in high-calcium medium for 0, 6, or 12 h and processed for immunoblotting analysis (Figure 3b). In control adenovirus-infected cells, the levels of keratin 1 and keratin 10 proteins were increased by 2-fold after differentiation. In Runx1- and Runx3-overexpressing keratinocytes, however, neither keratin 1 nor keratin 10 protein was detected by immunoblotting analysis before or after the shift to high-calcium conditions (note that the inhibition of keratin 10 expression by Runx3 was limited). Virus infection and the following immunoblot were performed three times and the band densities were measured. The levels of keratin 1 and keratin 10 proteins normalized relative to tubulin-alpha are shown as mean and SD (Supplemental Figure S4). In addition, the levels of *keratin 1* and *keratin 10* transcripts were decreased in Runx1- and Runx3-overexpressing keratinocytes, although the effects were more marked on *keratin 1* than *keratin 10* (Figure 3c). The results shown in Figure 3 suggest that Runx1 and Runx3 have inhibitory effects on the expression of *keratin 1* and *keratin 10*. These effects of Runx1/Runx3 on *keratin 1/keratin 10* were considered to be gene-specific, as overexpression of Runx1/Runx3 did not alter the levels of keratin 5 or keratin 14 expression, which are known to be expressed in both undifferentiated and differentiated keratinocytes (see Supplemental Figure S5).

### 2.4. Endogenous Runx1 and Runx3 Proteins Is Associated with the Promoter Sequences of Keratin 1 and Keratin 10 Genes in Undifferentiated but Not Differentiated Keratinocytes

**Figure 4:**
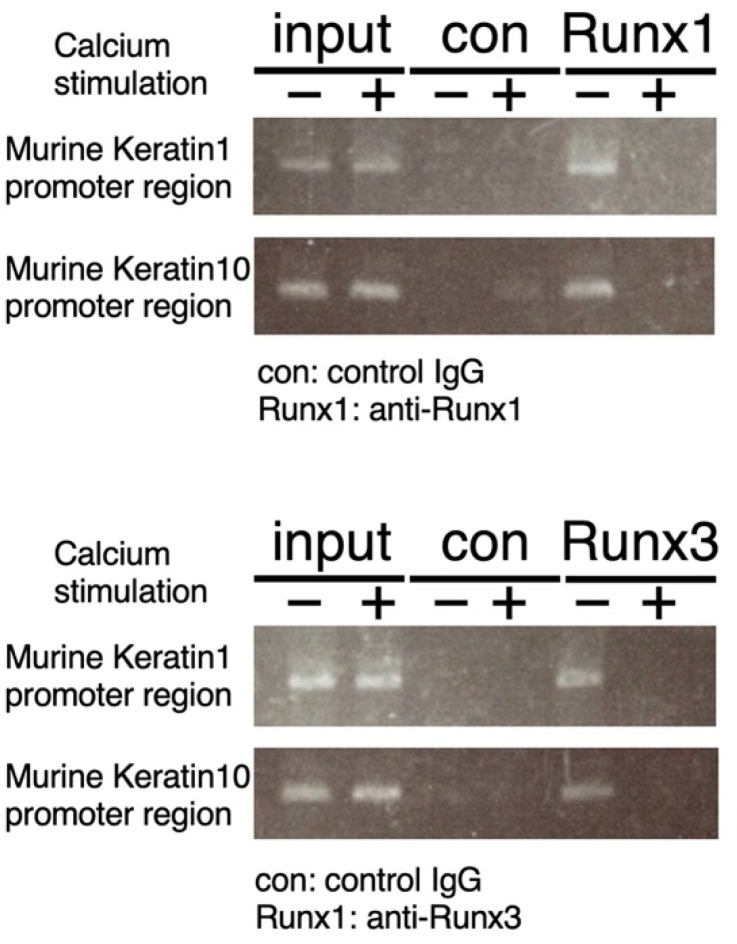
Chromatin immunoprecipitation (ChIP) of Runx1/Runx3 with *keratin 1* and *keratin 10* promoters. ChIP assay was performed to determine the binding of Runx1/Runx3 proteins to the *keratin 1* and *keratin 10* promoter sequences. The cells were cultured under low-calcium conditions (0 h) and high-calcium conditions (12 h), and cell lysates were prepared and processed for ChIP using anti-Runx1, anti-Runx3, or control IgG antibody. PCR was performed on the precipitated genomic DNA using primers flanking the Runx-binding consensus sites within the *keratin 1* promoter and *keratin 10* promoter. We performed three independent trials in the above experimental set out and could obtain essentially similar results. In the figure, representative results are shown.

The results discussed above suggest that endogenous Runx1 and Runx3 proteins directly regulate *keratin 1* and *keratin 10* gene transcription at least in undifferentiated keratinocytes. Therefore, we searched the *keratin 1* and *keratin 10* promoters and indeed found consensus sequences for Runx binding within the promoter regions of these genes (Supplemental Figure S6). We performed chromatin immunoprecipitation assays using antibodies specific for Runx1 and Runx3 and PCR primers for amplifying Runx consensus sites (Figure 4). Runx1 and Runx3 were found to be associated with *keratin 1* and *keratin 10* promoters in keratinocytes cultured in low-calcium medium but not in high-calcium medium. Therefore, it is possible that Runx1 and Runx3 directly suppress transcription of *keratin 1* and *keratin 10* in undifferentiated keratinocytes. The lack of Runx binding in differentiation-induced keratinocytes appeared reasonable, because Runx proteins were excluded from the nuclei, as shown in Figure 1b.

### 2.5. Inhibition of Runx1 and Runx3 Expression Did Not Alter Proliferation Capabilities of Cultured Keratinocytes

Undifferentiated keratinocytes are known to show active proliferation. We examined whether keratinocyte proliferation was influenced by Runx1 and/or Runx3. Endogenous *Runx1* and *Runx3* expression were abolished in cultured keratinocytes by siRNA treatment, as shown in Figure 2a, and the cellular incorporation of bromodeoxyuridine (BrdU) was measured. As shown in Supplemental Figure S7, inhibition of *Runx1* and *Runx3* expression did not significantly alter the level of BrdU incorporation. Therefore, Runx1 and Runx3 are likely not involved in the regulation of keratinocyte proliferation.

### 2.6. In Mouse Skin, Inhibition of Runx1 and Runx3 Expression Increased the Ratio of Keratin 1- and Keratin 10-Positive Keratinocytes in the Basal Layer of the Epidermis

**Figure 5:**
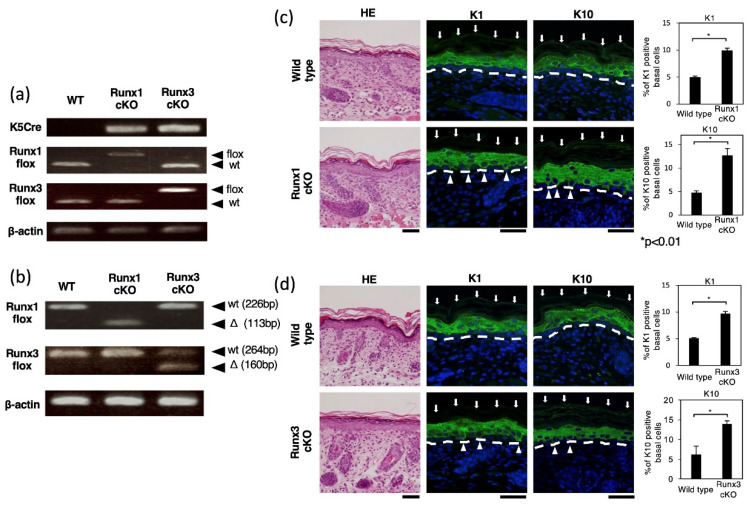
Effects of *Runx1/Runx3* knockout on keratin 1 and keratin 10 expression in keratinocytes. (**a**) Genotyping of the skin-specific *Runx1* or *Runx3* knockout mice. Genomic DNA was isolated from the tails of *K5Cre-Runx1fl/fl* mice, *K5Cre-Runx3 fl/fl* mice, and wild-type mice and processed for PCR. The alleles detected were *K5-Cre* transgene (**top** row), *Runx1* allele (**second** row), *Runx3* allele (third row), and *β-actin* (**bottom** row). (**b**) RT-PCR analysis of *Runx1* or *Runx3* transcripts in the epidermis-specific *Runx1* or *Runx3* knockout mice. RNA was isolated from the epidermis of *K5Cre-Runx1fl/fl* mice, *K5Cre-Runx3 fl/fl* mice, and wild-type mice and processed for RT-PCR. The detected transcripts were *Runx1* allele (**top** row), *Runx3* allele (**second** row), and *β-actin* (**bottom** row). Flox, floxed allele; wt, wild-type allele; Δ, deleted allele. (**c**,**d**) Immunostaining of keratin 1 and keratin 10 in the skin of epidermis-specific *Runx1* or *Runx3* knockout mice. Skin sections were prepared from *K5Cre-Runx1fl/fl* mice, *K5Cre-Runx3 fl/fl* mice, and wild-type mice and processed for staining with hematoxylin and eosin (H&E) and immunostaining for keratin 1 and keratin 10 (green fluorescence). Nuclei stained with DAPI are shown in blue. Arrows, skin surface; arrowheads, keratin 1-positive or keratin 10-positive basal keratinocytes; broken lines, dermal/epidermal junction. Scale bar, 50 μm. Six different visual fields were chosen randomly, using 40-fold magnification of optical lens and the numbers of keratin 1-positive and keratin 10-positive basal keratinocytes were counted (the total number of cells counted were roughly 1000). One of the authors (EO) prepared the tissue section and did immunostaining, whereas another author (TE), not informed of the mouse genotype of samples, counted the cell numbers through a microscope. The percentages of keratin 1-positive or keratin 10-positive cells per total epidermal cells are shown as the mean ± standard deviation. * *p* < 0.05. In (**c**,**d**), two different individual mice were used for each genotype and essentially similar results were obtained. In figure, representative results are shown.

In the epidermis, the basal keratinocytes represent undifferentiated cells that differentiate into spinous cells and then granular cells. Keratinocytes cultured under low-calcium conditions corresponded to basal keratinocytes, whereas those cultured in high-calcium medium corresponded to spinous and granular keratinocytes. We finally examined whether skin-specific inhibition of *Runx1* and *Runx3* expression affected keratin 1 and keratin 10 expression. We used *K5Cre*-transgenic mice [22], *Runx1-flox/flox* (*Runx1 fl/fl*) mice [23], and *Runx3*-*flox/flox* (*Runx3 fl/fl*) mice (these mice were generated in this study, see MATERIALS and METHODS section), which were crossed to obtain *K5Cre-Runx1 fl/fl* mice and *K5Cre-Runx3 fl/fl* mice. The respective genotype was confirmed by PCR analysis of the genomic DNA (Figure 5a), whereas inhibition of *Runx1* and *Runx3* expression in *K5Cre-Runx fl/fl* mice was confirmed by RT-PCR (Figure 5b).

Sections of mouse skin were prepared and processed for histological and immunohistochemical analyses (Figure 5c,d). Hematoxylin and eosin staining did not discern any particularly noticeable differences between wild-type and *Runx*-targeted mice in terms of cell morphology. On the other hand, immunohistochemical analysis revealed that the percentages of keratin 1- and keratin 10-positive cells in the basal layer of wild-type mouse skin was ~5%, whereas the corresponding values were increased by ~2-fold in the basal layer of *Runx1*- and *Runx3*-targeted mouse skin. Therefore, endogenous *Runx1* and *Runx3* appear to suppress the expression of keratin 1 and keratin 10 in the basal keratinocytes of the mouse epidermis.

It should be noted that the percentages of cells in the basal layer positive for the marker of cell proliferation, Ki67, were not significantly different between wild-type, *Runx1*-, and *Runx3*-targeted mouse skin (Supplemental Figure S8).

## 3. Discussion

In this study, we used primary cultures of mouse keratinocytes and demonstrated that both Runx1 and Runx3 proteins were expressed and localized in the nuclei of undifferentiated keratinocytes but translocated to the cytoplasm in differentiated keratinocytes. The siRNA-mediated inhibition of *Runx1* and *Runx3* expression increased keratin 1 and keratin 10 expression, whereas overexpression of Runx1 and Runx3 suppressed keratin 1 and keratin 10 expression. Endogenous Runx1 and Runx3 proteins were shown to be associated with the promoter sequences of *keratin 1* and *keratin 10* genes in the undifferentiated but not differentiated keratinocytes. In gene-targeted mice, inhibition of *Runx1* and *Runx3* increased the ratio of keratin 1- and keratin 10-positive cells in the basal layer of the epidermis. On the other hand, inhibition of Runx1 and Runx3 expression did not cause substantial alterations in the proliferation capacity of cultured or epidermal keratinocytes. Therefore, Runx1 and Runx3 possibly function to directly inhibit expression of *keratin 1* and *keratin 10* gene expression in undifferentiated keratinocytes, but are not involved in the regulation of keratinocyte proliferation.

A previous study reported that Runx1 protein was expressed in the nuclei of undifferentiated human keratinocytes, but its expression level was downregulated during cell differentiation [24]. This pattern of Runx1 expression was confirmed in both cultured keratinocytes and in the basal layer of the human epidermis. The same study further indicated, using cultured keratinocytes, that Runx1 binds to the promoter sequence of the keratin 1 gene and appears to activate its transcription, and that Runx1 functions to suppress the proliferation capacity of keratinocytes. The discrepancies between the results of the present study indicating that Runx1 inhibited *keratin 1* transcription, while Runx1 was reported previously to activate *keratin 1* transcription [24], may have been related to species-specific differences as this previous study used human cells/tissues and we used mouse cells/tissues. In addition, although Runx1 was reported to exert an inhibitory effect on cell proliferation, we could not reproduce such activity of Runx1 at least in siRNA experiments in the present study. We extended the possible role of Runx1 to that of Runx3 in the regulation of not only *keratin 1* but also *keratin 10* expression.

Considering their functions as transcription factors, Runx proteins are expected to be localized to the nucleus. It should be noted that cytoplasmic localization of Runx has also been reported in some cases [15]. This is particularly intriguing in that nuclear Runx3 is expected to exert an anti-oncogenic effect and its sequestration into the cytoplasm may contribute to oncogenesis. Our results suggested that nuclear-localized Runx1/Runx3 in undifferentiated cells was capable of binding to the promoter sequence and inhibits *keratin 1/keratin 10* transcription, whereas cytoplasmic-located Runx1/Runx3 in differentiated cells had lost both promoter sequence binding ability and the inhibitory effect on *keratin 1/keratin 10* expression. Thus, in our model, the differentiation-induced expression of keratin 1/keratin 10 was apparently a consequence of release from transcriptional inhibition due to the translocation of Runx protein.

In the field of keratinocytes’ physiology, roles of p63 transcription factor and Notch signaling molecule have been extensively investigated. On the other hand, reports on possible functions of Runx transcription factors have been very limited, and the present study is actually the first case reporting some activity of Runx3 in keratinocytes. From a pathological aspect, a genome-wide association study (GWAS) suggested the possible involvement of Runx1 and Runx3 in psoriasis, an abnormal manifestation of keratinocyte proliferation and differentiation [25,26]. In addition, altered expression of Runx1 has been reported for non-melanoma skin malignancies [24]. Thus, further investigations are necessary to determine the implications of the present findings together with those of previous studies for understanding the pathology of diseases involving keratinocytes.

## 4. Materials and Methods

### 4.1. Cell Culture

Primary cultures of keratinocytes were prepared from the epidermis of newborn ICR mice as described previously [27]. Briefly, the epidermis was separated from the dermis and incubated in 0.25% (*w*/*v*) trypsin solution (Gibco BRL, Tokyo, Japan) overnight at 4 °C. The dispersed cells were plated in dishes coated with collagen type I (Nitta Gelatin, Osaka, Japan), and cultured in minimal essential medium (MEM) supplemented with 4% (*v*/*v*) Chelex (Bio-Rad, Hercules, CA, USA)-treated fetal calf serum (FCS), epidermal growth factor (10 ng/mL; Gibco BRL), and 0.05 mM CaCl2. Under these conditions, keratinocytes were maintained in an immature and proliferating state. The cells were used for experiments 1 week after plating.

To induce differentiation of keratinocytes, CaCl_2_ concentration in the medium was increased to 0.2 mM. Calcium treatment induced morphological changes in cells as observed through a phase-contrast microscope and also induced expression of filaggrin protein, a marker of keratinocytes differentiation, as detected by immunoblot (Supplemental Figure S9). The cells were incubated in a medium containing 10 μM BrdU for 5 h, permeabilized with 0.1% (*v*/*v*) NP-40, and denatured in 50 mM NaOH. BrdU incorporation was detected using an anti-BrdU antibody (BD Biosciences, Franklin Lakes, NJ, USA). The nuclei were stained with DAPI (Invitrogen, Carlsbad, CA, USA).

### 4.2. RNAi and Recombinant Adenovirus Infection

The siRNAs targeting *Runx1* or *Runx3* were purchased from Invitrogen. Their sequences were as follows: for *Runx1*-siRNA1, 5′-AAGAGGUGAUGGAUCCCAGGUACUGCAGUACCUGGGAUCCAUCACCUCUU-3′; for *Runx1*-siRNA2, 5′-UAUAGAUGGUAGGAGGGCGAGCCGGCCGGCUCGCCCUCCUACCAUCUAUA-3′; for *Runx3*-siRNA1, 5′-ACGAAUCGAAGGUCGUUGAACCUGGCCAGGUUCAACGACCUUCGAUUCGU-3′; and for *Runx3*-siRNA2, 5′-UUGUGAGCGUGAAACUCUUCCCUCGCGAGGGAAGAGUUUCACGCUCACAA-3′. Cells were transfected with siRNAs using Lipofectamine 2000 (Invitrogen). Block-iT Fluorescent Oligo (Invitrogen) showed that siRNAs were introduced into more than 80% of transfected cells. Recombinant adenoviruses expressing either *Runx1*, *Runx3*, or *LacZ* were constructed as described previously [4]. Cultured keratinocytes were infected with virus at a multiplicity of infection (moi) of 50.

### 4.3. Antibodies

The following antibodies were purchased and used as recommended by their suppliers: rabbit polyclonal antibodies against keratin 1, keratin 5, keratin 10, and keratin 14 (Covance, Emeryville, CA, USA); mouse anti-α-tubulin monoclonal antibody and mouse anti-β-actin monoclonal antibody (Sigma, St. Louis, MO, USA); rabbit anti-Ki67 polyclonal antibody (Novocastra, Wetzlar, Germany); rabbit anti-Runx1 monoclonal antibody, rabbit anti-Runx3 monoclonal antibody (MBL, Tokyo, Japan); and fluorescein isothiocyanate (FITC)-conjugated and horseradish peroxidase-conjugated goat anti-mouse and goat anti-rabbit IgG antibodies (Amersham, Tokyo, Japan). Secondary antibodies conjugated with Alexa Fluor dyes were purchased from Life Technologies (Thermo Fisher, Waltham, MA, USA).

Anti-panRunx antibody was described previously [28].

### 4.4. Immunostaining of Cells and Tissues

Cultured keratinocytes were fixed with 4% (*w*/*v*) paraformaldehyde solution and incubated with antibody against Runx1 or Runx3 followed by FITC-conjugated secondary antibody. The nuclei were stained with DAPI. Stained preparations were photographed with a TCS 4D scanner connected to an inverted microscope (LEITZ DM IRB; Leica Camera AG, Wetzlar, Germany).

Isolated mouse skin was cut into frozen sections 6 μm thick and fixed with 2% paraformaldehyde. The sections were preincubated in 5% (*v*/*v*) serum for 30 min and incubated with primary antibodies followed by secondary antibodies. Stained preparations were observed with an LSM 5 exciter (Zeiss, Jena, Germany).

### 4.5. Immunoblotting

Proteins were extracted from the cells with 2× Laemmli sample buffer (125 mM Tris, pH 6.8, 4% (*w*/*v*) SDS, 20% (*v*/*v*) glycerol, 5% (*w*/*v*) β-mercaptoethanol), separated by SDS–polyacrylamide gel electrophoresis and transferred onto Immobilon-P membranes (Millipore, Billerica, MA, USA). Blots were incubated with primary antibodies and horseradish peroxidase-conjugated secondary antibodies and developed by the ECL detection system (Cell Signaling Technology, Danvers, MA, USA).

### 4.6. Reverse Transcription Followed by Semi-Quantitative or Real-Time PCR (RT-PCR)

Total cytoplasmic RNA was isolated from the cells/tissues using TRIzol reagent (Invitrogen). The cDNA was synthesized from RNA templates using Moloney murine leukemia virus-derived reverse transcriptase (Promega, Madison, WI, USA). Primers for detecting proximal or distal *Runx1/Runx3* transcripts were purchased from Eurofins Genomics (Tokyo, Japan), whereas primers for *keratin 1*, *keratin 10*, and *hypoxanthine phosphoribosyltransferase* (*HPRT*) transcripts were from Applied Biosystems (Foster City, CA, USA). Primers for *glyceraldehyde-3-phosphate dehydrogenase* (*GAPDH*) were from Invitrogen. To detect *Runx1*, *Runx3*, and *GAPDH* transcripts, semi-quantitative PCR was performed using a StepOne™ RT-PCR System (Applied Biosystems) and a Universal Probe Library Set (Roche Applied Science, Indianapolis, IN, USA). To detect *keratin 1*, *keratin 10*, and *HPRT* transcripts, real-time PCR was performed using TaqMan Fast Advanced Master Mix on a StepOne RT-PCR System (Applied Biosystems). PCR conditions were as follows: for *Runx1*, 39 cycles of 95 °C for 15 s, 58 °C for 30 s, and 72 °C for 40 s; for *Runx3*, 40 cycles of 94 °C for 30 s, 55 °C for 30 s, and 72 °C for 40 s; for *GAPDH*, 35 cycles of 94 °C for 30 s, 55 °C for 30 s, and 72 °C for 40 s; for *keratin 1*, *keratin 10*, and *HPRT*, 40 cycles of 95 °C for 1 s, 60 °C for 20 s. The cDNA quantity in each sample was normalized relative to *HPRT* as a control.

### 4.7. Chromatin Immunoprecipitation

Chromatin immunoprecipitation was performed as described previously [29]. Briefly, cultured keratinocytes (5 × 10^5^) were crosslinked with 1% (*w*/*v*) formaldehyde (Sigma) in PBS for 30 min and lysed in PBS containing 1% (*w*/*v*) SDS. Lysates were sonicated to fragment chromatin DNA, preincubated with Dynabeads Protein G and Protein A (Invitrogen), and centrifuged. To the supernatants were added anti-Runx1 antibody (Abcam, Cambridge, UK), anti-Runx3 antibody (3F12), or control mouse immunoglobulin (Santa Cruz Biotechnology, Santa Cruz, CA, USA) and incubated overnight at 4 °C. Immune complexes were absorbed to Dynabeads Protein G and Protein A, and protein crosslinking was reverted by heating the mixture at 65 °C for at least 8 h. The mixture was then incubated in 1% (*w*/*v*) SDS, 0.1 M NaHCO3 and treated with RNase A and proteinase K. DNA was purified using a QIAquick PCR-Purification Kit (QIAGEN, Hilden, Germany). PCR was performed with the recovered DNA as the template using primers for *keratin 1* or *keratin 10* promoter sequences as follows: *keratin 1* forward, 5′-ACA ATA CCC TAG TGA GTG TGT GGG C-3′, *keratin 1* reverse, 5′-AGG GTT TGG CTC GCC TGC AGC CAT AC-3′; *keratin 10* forward, 5′-CCT TGA AGA ACC TCA GTC TGG-3′, and *keratin 10* reverse, 5′-TTT AGG AGA CCA CTG AAG GCC-3′.

### 4.8. Generation of Runx3-Floxed Mice

The Runx3-floxed mice (Accession No. CDB0458K: https://large.riken.jp/distribution/mutant-list.html, accessed on 30 May 2022) were generated by conventional gene targeting method as follows. As shown in Supplemental Figure S10a, the DNA fragments corresponding to the 5′ and 3′ sequences of *Runx3* exon 4 were obtained from the corresponding BAC clones by digestion with the appropriate restriction enzymes. The genomic fragments, as well as the *loxP* site, *frt*-flanked *neomycin resistance* cassette, and *diphtheria toxin subunit A* gene, were inserted into the targeting vector. The resulting plasmid DNA was linearized and electrophoretically transfected into TT2 ES cells [30], which were derived from an F1 of a C57BL/6 × CBA mouse mating. Positive and negative selection and PCR genotyping yielded eight colonies of embryonic stem (ES) cells. Recombination was verified by Southern blotting analysis using *Runx3* and *neomycin* probes (Supplemental Figure S10b). Three colonies (No. 35, 77, and 109) were injected into 8-cell stage embryos of CD-1 mice and were successfully transmitted through the germ line. *Runx3* heterozygous mice derived from two clones (35 and 109) were then backcrossed to C57BL/6 mice for more than 10 generations, and during these matings heterozygous mice were crossed with *flp-frt* mice [31] resulting in deletion of the neomycin resistance cassette. The mice thus obtained were designated as *Runx3* (*flox*/*flox*) or *Runx3* (*flox*/*wt*). The primers to identify the genotypes of the *Runx3 floxed* allele and wild-type allele were as follows: forward, 5′-GAA CCC CGA CGT AAG TGC TAC TGA G-3′ and reverse, 5′-TGC AGA GCA GAC TGG CCT AAG CCT C-3′. These primers span exon 4 of the *Runx3* gene. The sizes of PCR-amplified products were 267 bps and 415 bps, respectively, for the wild type and floxed allele of *Runx3*.

### 4.9. Generation of Keratinocyte-Specific Gene-Deletion Mice

*Keratin5 (K5)-Cre* transgenic mice and *floxed*-*Runx1* mice were described previously [22,23]. To generate knockout mice, we mated homozygous *K5-Cre* males with *Runx1 (flox*/*flox)* or *Runx3 (flox*/*flox)* females to generate *K5-Cre/Runx1 (flox*/*wt)* or *K5-Cre*/*Runx3 (flox*/*wt)* mice. F1 mice genotyped as *K5-Cre/Runx1 (flox*/*wt)* or *K5-Cre/Runx3 (flox*/*wt)* were crossed with *K5-Cre/Runx1 (flox*/*wt)* or *K5-Cre*/*Runx3 (flox*/*wt)* mice to obtain F2 mice that would be genotyped as *K5-Cre/Runx1 (flox*/*flox)* or *K5-Cre*/*Runx3 (flox*/*flox)*. Mouse genomic DNA was isolated from tail biopsies using a DNeasy Blood and Tissue kit (QIAGEN). The primers used to identify the genotype, such as *K5-Cre* and *Runx1 floxed* allele and wild-type allele, were as described above.

### 4.10. Statistical Analysis

The quantitative data were analyzed using the unpaired Student’s *t* test. In all analyses, *p* < 0.05 was taken to indicate statistical significance.

## Data Availability

Not applicable.

## Supplementary Materials

The following supporting information can be downloaded at: https://www.mdpi.com/article/10.3390/ijms231710039/s1.
