# Supplementary material for: Transcription Factors Runx1 and Runx3 Suppress Keratin Expression in Undifferentiated Keratinocytes
Source: Int J Mol Sci. 2022 Sep 2;23(17):10039. doi: 10.3390/ijms231710039 (PMC9456233; doi:10.3390/ijms231710039)
Supplement: Supplementary file 1 [file ijms-23-10039-s001.zip › ijms-1792200-supplementary.pdf]

International Journal of
Molecular Sciences

MDPI

LEGENDS FOR SUPPLEMENTAL FIGURES

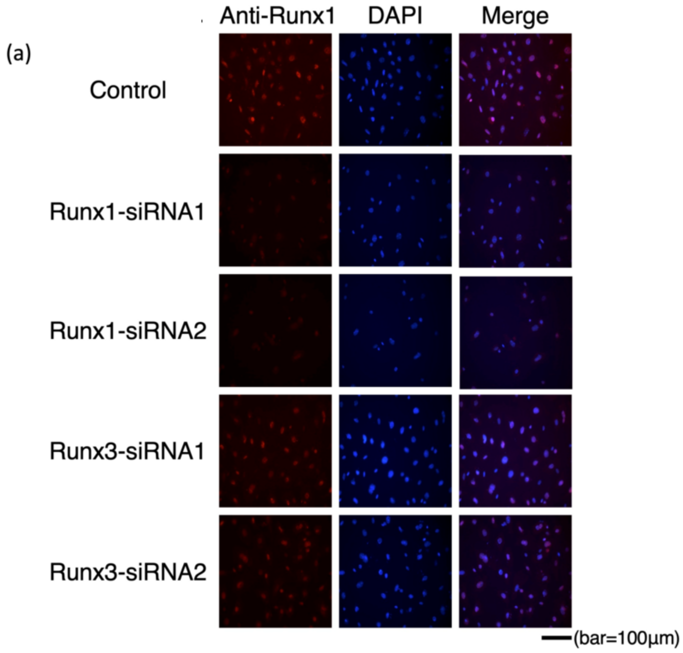

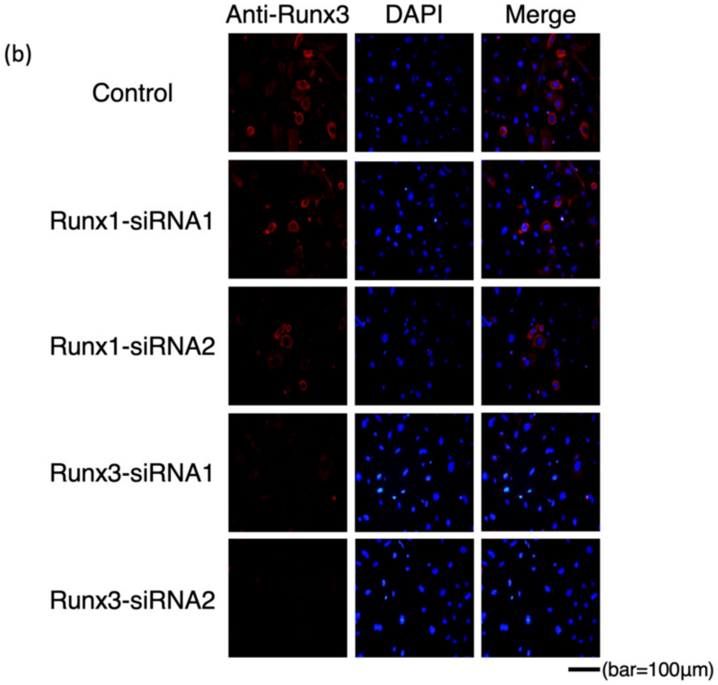

Figure S1. Effects of *Runx1*/*Runx3* siRNAs on Runx1/Runx3 protein expression in cultured keratinocytes. The cultured keratinocytes were treated by the indicated si-RNA for 4h, fixed and processed for immunostaining using the Runx1- and Runx3-specific antibodies, respectively. In the *Runx1*-siRNA-treated cells, the staining intensity of Runx1 (but not of Runx3) was markedly reduced, whereas in the *Runx3*-siRNA-treated cells the staining intensity of Runx3 (but not of Runx1) was markedly reduced.

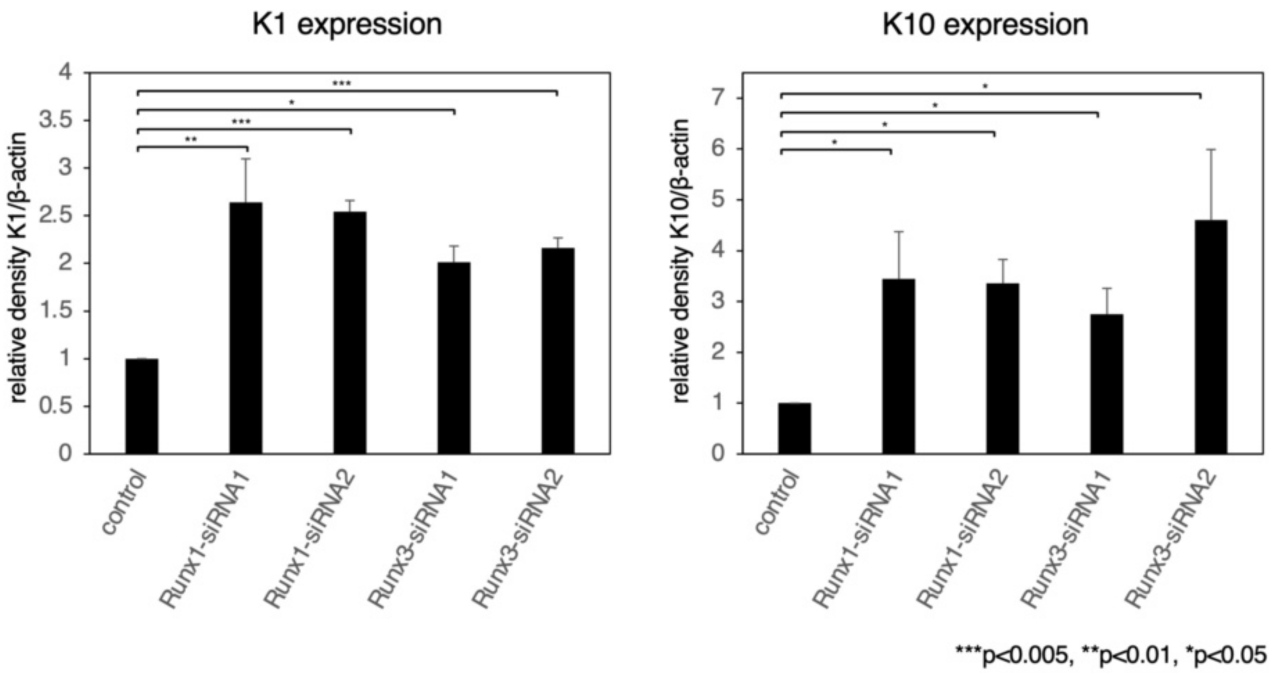

Figure S2. Effects of *Runx1*/*Runx3* siRNAs on keratin 1 and keratin 10 protein expression in cultured keratinocytes. The levels of keratin 1 and keratin 10 proteins normalized relative to beta-actin are shown as mean ± standard deviation. Statistically significant differences, if detected, are indicated by brackets (a student t-test).

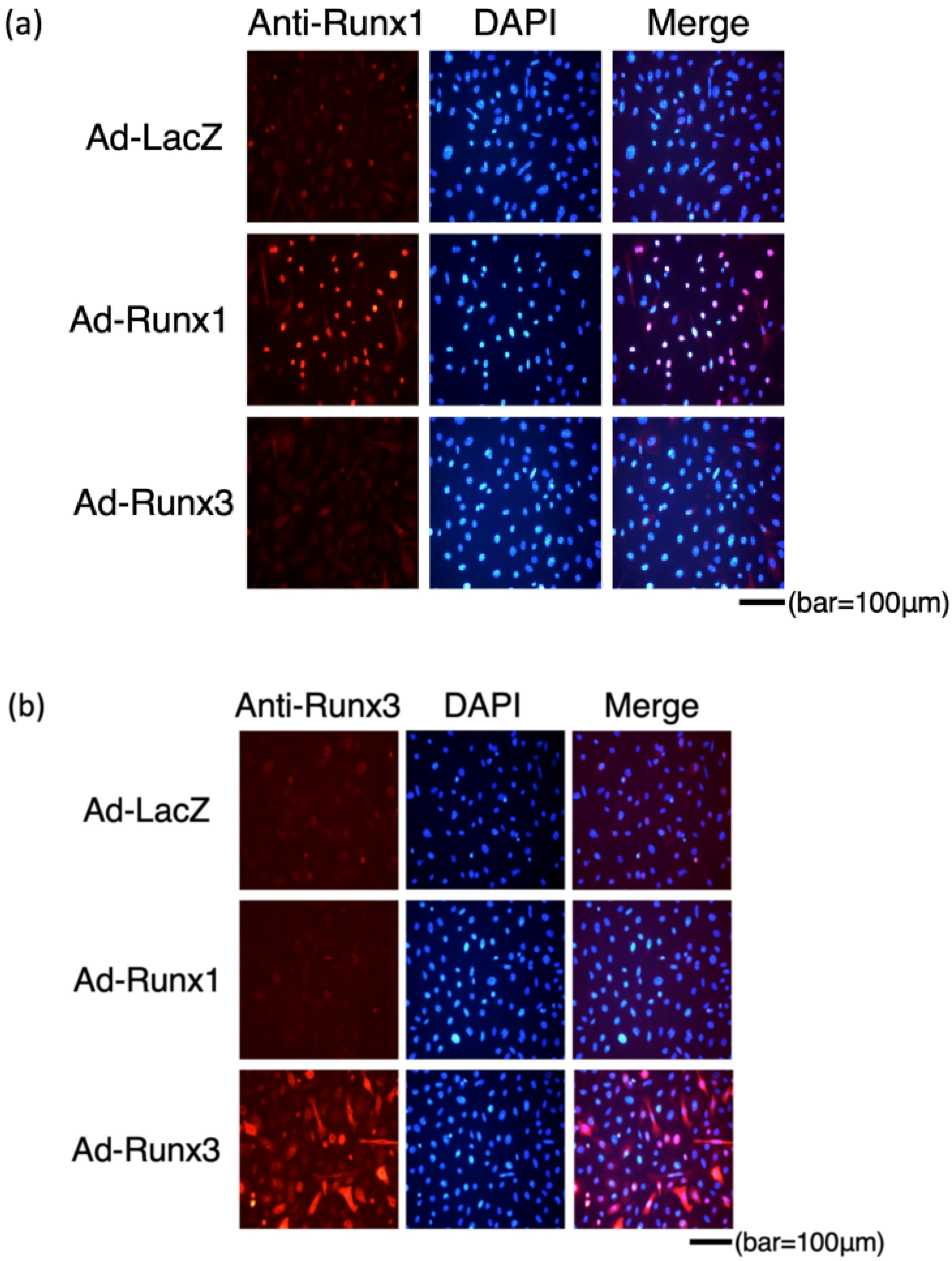

Figure S3. Detection of overexpressed Runx proteins by immunostaining. The cultured keratinocytes were infected by adenovirus-*LacZ*, adenovirus-*Runx1* and adenovirus-*Runx3*, respectively, incubated for 24 h, fixed and processed for immunostaning using the Runx1- and Runx3-specific antibodies.

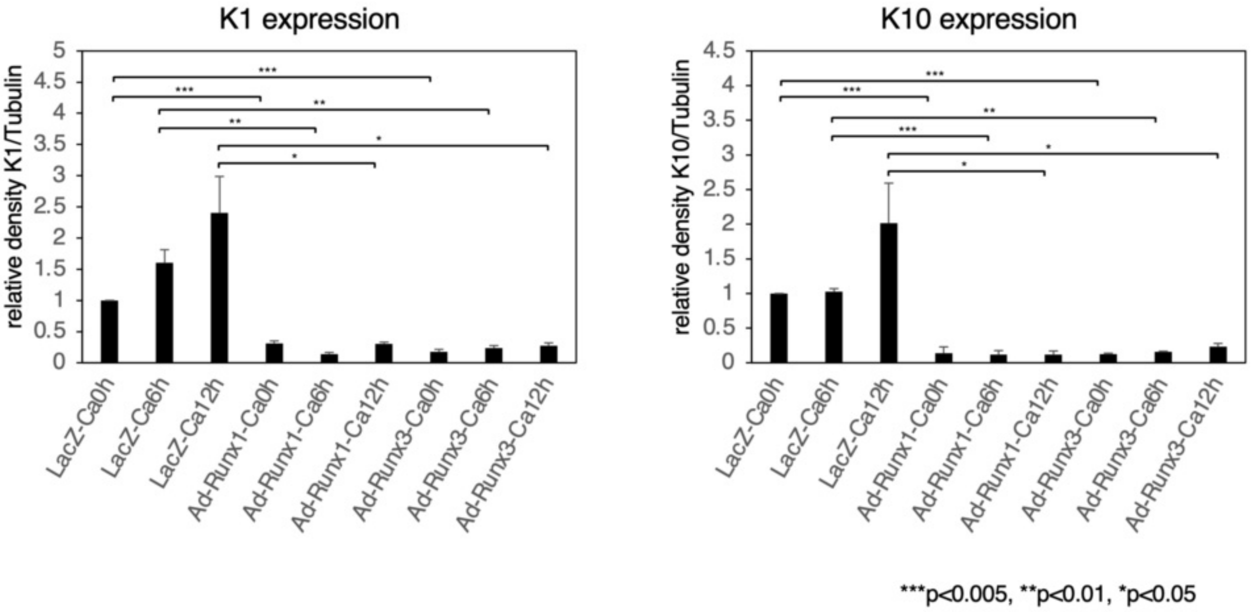

Figure S4. Effects of Runx1/Runx3 overexpression on the keratin 1 and keratin 10 protein levels in cultured keratinocytes. The levels of keratin 1 and keratin 10 proteins normalized relative to tubulin-alpha are shown as mean ± standard deviation. Statistically significant differences, if detected, are indicated by brackets (a student t-test).

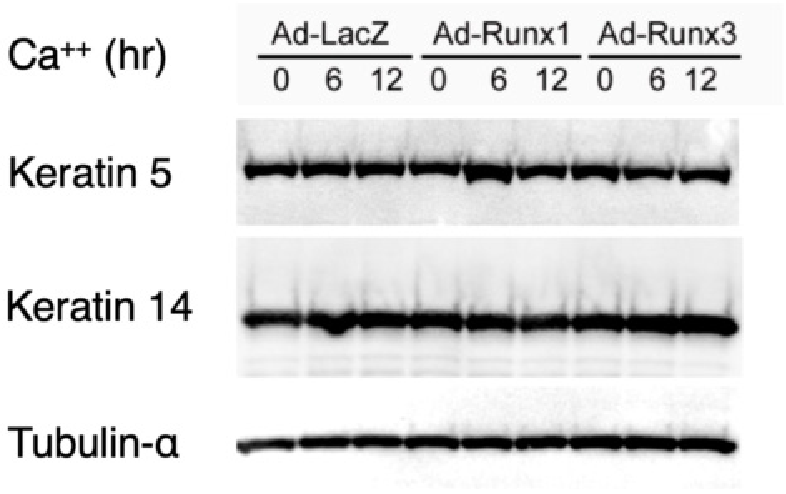

Figure S5. Effects of Runx1/Runx3 overexpression on the keratin 5 and keratin 14 protein levels in cultured keratinocytes. Experiments were essentially identical to those in Figure 3b except that keratin 5 and keratin 14 expression were examined by immunoblotting. Experiments were performed three times and essentially similar results were obtained.

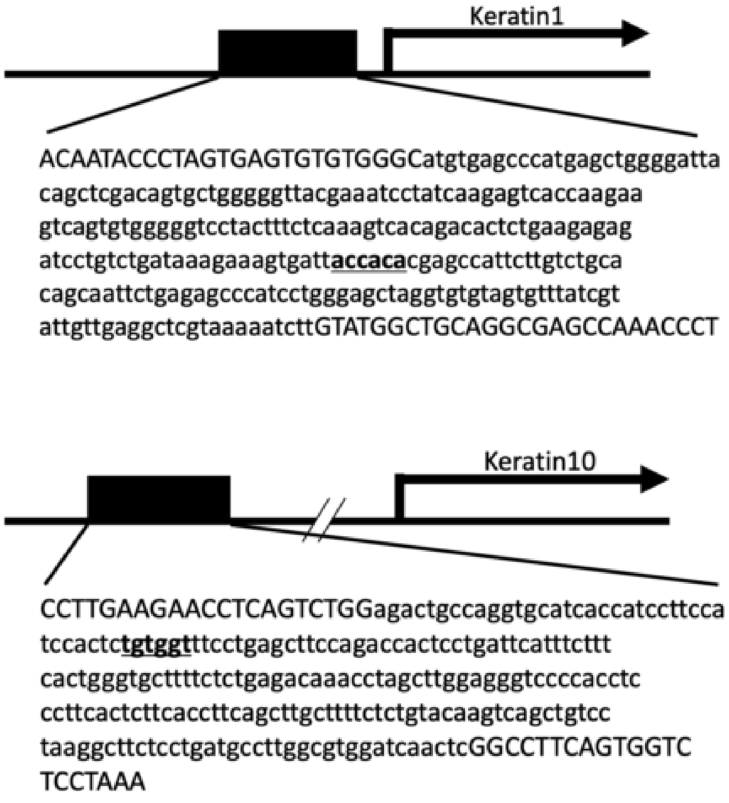

Figure S6. Runx binding consensus sites (bold and underlined) in the promoter sequences of mouse *keratin 1* and *keratin 10* genes. The sequences indicated in upper case correspond to the primers used for ChIP analysis.

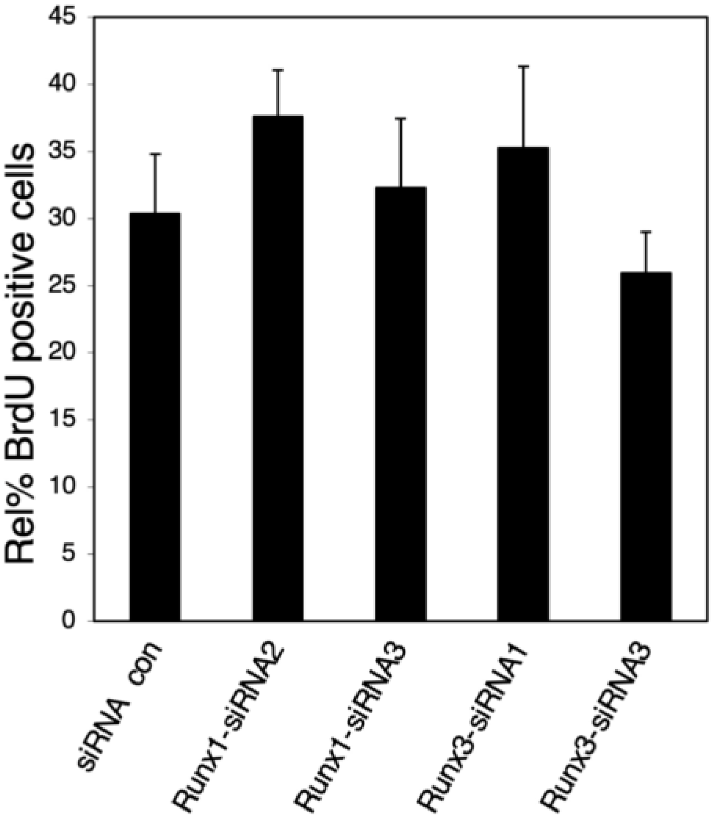

Figure S7. Effects of inhibition of *Runx1*/*Runx3* expression on proliferation of cultured keratinocytes. The cells were transfected with siRNA specific to *Runx1* or *Runx3*, incubated for 4 h, induced to differentiate by raising the calcium concentration for 12 h, incubated in medium containing BrdU for the last 5 h, and harvested. Since the total 16 h were relatively short interval, cell proliferation was evaluated not by counting the cell number but by measuring the BrdU incorporation. The mean ± SD percentages of BrdU-positive cells are shown. Experiments were performed three times and essentially similar results were obtained.

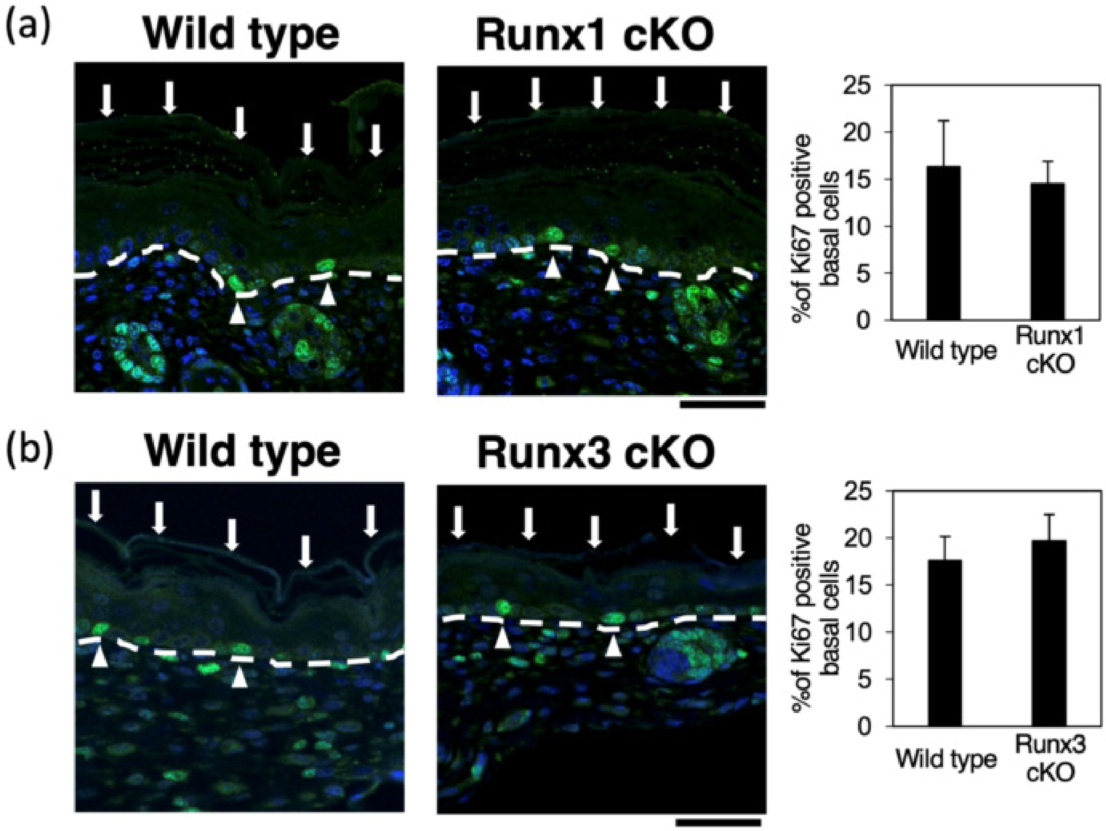

Figure S8. Effects of skin-specific *Runx1*/*Runx3* knockout on the expression of Ki67 in mouse skin. The experiments were essentially identical to those in Figure 5c and d except that expression of Ki-67 was examined instead of keratin 1 and keratin 10 by immunostaining. Two different individual mice were used for each genotype and essentially similar results were obtained.

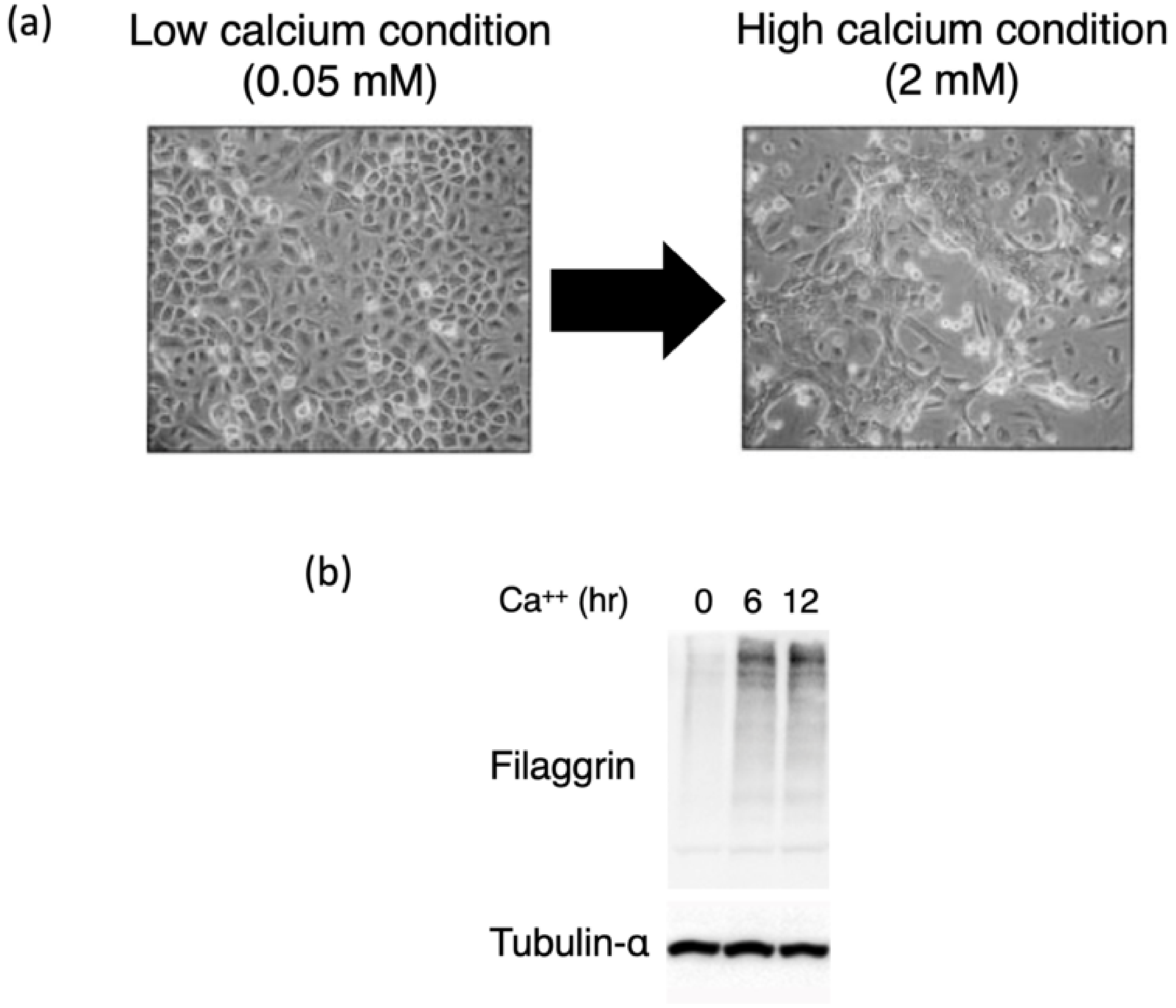

Figure S9. Calcium-induced keratinocytes' differentiation. (a) Phase-contrast microscopic images of keratinocytes in low and high calcium medium. (b) Calcium-induced expression of filaggrin, a marker of keratinocytes' differentiation, as detected by immunoblot.

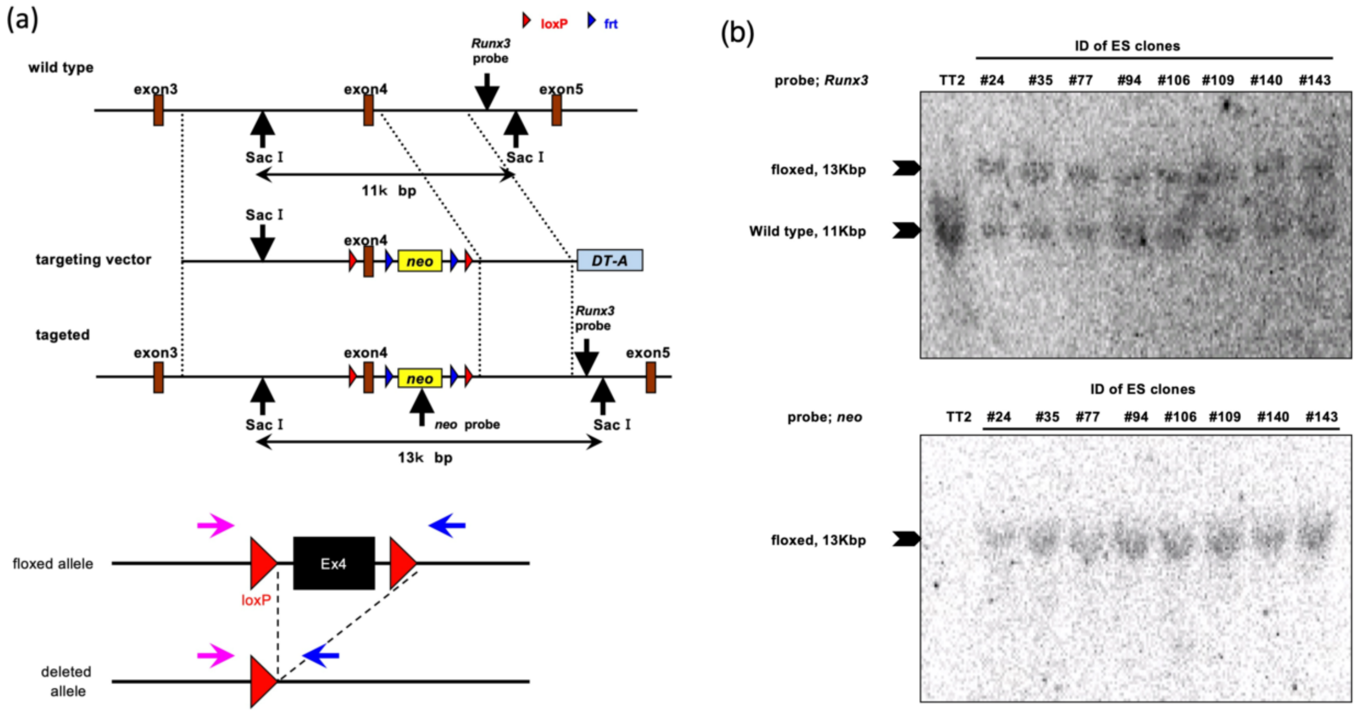

Figure S10. Generation of *Runx3*-targeted ES cell clones. (a) Physical maps of the *Runx3* gene locus and its targeting vector. The features of the wild-type as well as targeted *Runx3* alleles and features of the targeting vector are depicted. Horizontal lines indicate the genomic sequences. Exons 3, 4, and 5 of the *Runx3* gene, *neomycin resistance* cassette, and *diphtheria toxin subunit A* gene are indicated. Red and blue rectangles indicate the *loxP* and *frt* sequences, respectively, whereas triangles indicate *Runx3* and *neomycin* probes and *Sac*I restriction sites used for Southern blotting. (b) Southern blotting analysis of genomic DNA prepared from ES cell clones. Eight ES cell colonies were obtained by transfecting parental ES cells, TT2, with the targeting vector and selecting the surviving cells. The ID numbers of colonies are indicated. DNA was digested with *Sac*I and processed for Southern blotting using the probes for *Runx3* (upper panel) and *neomycin* (lower panel). The sizes of detected bands for the wild-type and floxed alleles are indicated in kilobase pairs (kb).
